# Comparison of passive-scattered and intensity-modulated proton beam therapy of craniospinal irradiation with proton beams for pediatric and young adult patients with brain tumors

**DOI:** 10.1007/s11604-023-01499-8

**Published:** 2023-10-24

**Authors:** Nobuyoshi Fukumitsu, Hikaru Kubota, Yusuke Demizu, Takeshi Suzuki, Daiichiro Hasegawa, Yoshiyuki Kosaka, Atsufumi Kawamura, Toshinori Soejima

**Affiliations:** 1 Department of Radiation Oncology, Kobe Proton Center, 1-6-8, Minatojima-Minamimachi, Kobe, 650-0047 Japan; 2 Department of Anesthesiology, Kobe Proton Center, Kobe, Japan; 3 Department of Hematology and Oncology, Hyogo Children’s Hospital, Kobe, Japan; 4 Department of Neurosurgery, Hyogo Children’s Hospital, Kobe, Japan

**Keywords:** CSI, PBT, PSPT, IMPT

## Abstract

**Purpose:**

To investigate the dose stability of craniospinal irradiation based on irradiation method of proton beam therapy (PBT).

**Methods and materials:**

Twenty-four pediatric and young adult brain tumor patients (age: 1–24 years) were examined. Treatment method was passive-scattered PBT (PSPT) in 8 patients and intensity-modulated PBT (IMPT) in 16 patients. The whole vertebral body (WVB) technique was used in 13 patients whose ages were younger than 10, and vertebral body sparing (VBS) technique was used for the remaining 11 patients aged 10 and above. Dose stability of planning target volume (PTV) against set-up error was investigated.

**Results:**

The minimum dose (*D*_min_) of IMPT was higher than that of PSPT (*p* = 0.01). Inhomogeneity index (INH) of IMPT was lower than that of PSPT (*p* = 0.004). When the irradiation field of the cervical spinal cord level (C level) was shifted, the maximum dose (*D*_max_) was lower in IMPT, and mean dose (*D*_mean_) was higher than PSPT as movement became greater to the cranial–caudal direction (*p* = 0.000–0.043). *D*_min_ was higher and INH was lower in IMPT in all directions (*p* = 0.000–0.034). When the irradiation field of the lumber spinal cord level (L level) was shifted, *D*_max_ was lower in IMPT as movement became greater to the cranial direction (*p* = 0.000–0.028). *D*_min_ was higher and INH was lower in IMPT in all directions (*p* = 0.000–0.022).

**Conclusions:**

The PTV doses of IMPT and PSPT are robust and stable in both anterior–posterior and lateral directions at both C level and L level, but IMPT is more robust and stable than PSPT for cranial–caudal movements.

**Trial registry:**

Clinical Trial Registration number: No. 04-03.

## Introduction

Craniospinal irradiation (CSI) is the standard treatment for brain tumors prone to leptomeningeal dissemination, such as pediatric medulloblastoma and some germ cell tumors [1, 2]. Owing to the large field size, a significant volume of normal organs is included in the irradiated area [3]. Therefore, various adverse effects, including hematological and gastrointestinal toxicities, are observed during and after CSI [4–8]. In an effort to reduce the potential toxicities related to CSI, advanced techniques have been adopted. Among these, proton beam therapy (PBT) has received attention due to its dosimetric advantage [9, 10]. In contrast to photon radiotherapy (RT), PBT involves a sharp rise and fall in energy deposition, known as the Bragg peak, which stops at the end of the finite beam range due to its physical feature [11]. Therefore, PBT can reduce unnecessary dose to the neck, chest, and abdominal organs [11–13], and fewer acute and late adverse effects are expected in PBT compared to photon RT.

Techniques for the delivery of PBT have advanced in recent decades. One of the most representative advances is the development of the spot scanning technique using pencil beams [14]. In the spot scanning irradiation technique, a lesion is visualized as a mass of points, and each point is irradiated individually, unlike in conventional passive-scattered broad-beam irradiation, in which a bundle of proton beams that are shaped to match the lesion is used. Scanning PBT is associated with superior beam flexibility that allows adaptation to complex-shaped targets. It can easily accomplish superior target coverage and further reduction of normal tissue irradiation than passive-scattered PBT (PSPT) using technique such as intensity-modulated PBT (IMPT). In PSPT, a constant dose is delivered within the irradiation field. The beam spreads out in a fan shape, there is a gap proximal to the irradiation port between the beam and the neighboring beam, and the beams overlap distally. IMPT can theoretically adjust the dose distribution to be not too much or not too little by applying a gradient to the dose at the field junction. Other advantages are the reduced cost of manufacture of patient-specific apertures or compensators and the reduced time needed during delivery to change the devices [15, 16]. The number of facilities offering IMPT is growing rapidly worldwide. The advantage of IMPT for the treatment of CSI is expected as normal tissue dose reduction and target dose robustness. However, CSI using PBT was performed and investigated with very small number of patients (less than 10) [17–19], and it is difficult to determine the effects of technical differences in irradiation techniques.

Our facility has been established in Dec 2017 adjacent to one of the largest pediatric cancer regional core hospitals in our country. The number of treated pediatric patients using PBT has been the highest since 2018 in our country. We compared robustness of planning target volume (PTV) dose based on irradiation technique of PBT in pediatric and young adult brain tumor patients who received CSI in our facility.

## Materials and methods

All the study procedures involving human participants were conducted in accordance with the ethical standards of the institutional research committee, in compliance with the Declaration of Helsinki, and with the approval of the institutional review board (No. 04-03). This study was conducted as a retrospective study, and we obtained patient consent via the opt-out method using the hospital’s website. The patients were 24 consecutive patients with pediatric and young adult brain tumor patients who completed CSI between Feb 2019 and Mar 2021; age: 1–24 (median 10) years). Primary diseases were medulloblastoma: 18, germ cell tumors: 3, choroid plexus tumor: 1, undifferentiated large cell lymphoma: 1 and glioma: 1 patient. RayStation, version 7 or 9 (RaySearch Medical Laboratories, Stockholm, Sweden) was used for treatment planning. The CTV included whole brain and spinal canal. Planning target volume (PTV) was set to CTV + 3 mm in the brain and CTV + 6 mm in the spinal canal. The vertebral bodies were included in the PTV in 13 patients whose age was younger than 10. Treatment method was PSPT in 8 patients (Feb–Oct 2019) and IMPT in 16 patients (Jan 2020–Mar 2021). As the field size was 15 × 20 cm in our facility, it was not possible to irradiate the entire PTV with one irradiation field. Thus, the PTV were divided in the SI direction. The number of divisions varied dependent on patients’ height. Brain beam direction was two opposing left and right in six cases and two oblique posterolateral in two cases in PSPT, and two oblique posterolateral in all cases in IMPT. The direction of the spinal cord beam was one posterior beam in both PSPT and IMPT. To mitigate hot and cold spots at the field junction, two sets of plans with different levels of the field were used in PSPT. For the patients of IMPT, each irradiation field was joined by sharing a 7 cm junction area with a linear slope in the dose distribution. Robust setting was 1% of range uncertainty to the PTV in the brain and 4 mm to the superior and inferior direction with 1% of range uncertainty to the PTV in the spinal cord levels. Total irradiation dose of CSI was 18 Gray (relative biological effect) (Gy(RBE)): 3, 23.4 Gy(RBE): 17, 25.2 Gy(RBE): 2, 36 Gy(RBE): 2, all 1.8 Gy(RBE) daily. The dose constraint for CTV is between 90 and 105%, and the acceptable range is between 83 and 106%. In addition, we try as much as possible to keep the PTV dose constraint between 90 and 105%. Cases in which dose constraints cannot be met are determined on a case-by-case basis. Boost irradiation to the local region was performed in 23 of 24 patients. Neoadjuvant and concurrent chemotherapy was performed in 23 and 19 patients, respectively. Follow-up period was 17–44 (median 27) months as of Dec 2022.

We investigated PTV dose stability. We conducted a simulation to reproduce the situation in which the irradiation fields moved by shifting the iso-center coordinates of the irradiation field of cervical and lumber spinal cord level (C and L levels) step by step on the treatment planning system. The C and L levels were shifted up to a maximum of 5 mm by 1 mm to the antero-posterior, lateral, and cranial–caudal directions, respectively. We calculated 5 parameters for the PTV: the maximum dose (*D*_max_), mean dose (*D*_mean_), minimum dose (*D*_min_), conformity index (CI), and inhomogeneity index (INH). CI and INH were calculated as follows:

$$\mathrm{CI }= \frac{{\mathrm{PTV}}_{\mathrm{pre}}}{\mathrm{PTV}}\times \frac{{\mathrm{PTV}}_{\mathrm{pre}}}{{V}_{\mathrm{pre}}},$$

$$\mathrm{INH}\hspace{0.17em}=\hspace{0.17em}\frac{{D}_{2}-{D}_{98}}{{D}_{\mathrm{pre}}},$$

where PTV_pre_ is the PTV covered by the prescription dose, *V*_pre_ is the volume of the prescription isodose, *D*_2_ and *D*_98_ are the doses to 2 and 98% of the PTV, and *D*_pre_ is the prescription dose.

The data represent mean value and standard deviation. Unpaired *t* test was used for comparing the data between the patient groups, and *p* values of less than 0.05 were considered as being indicative of statistical significance.

## Results

The number of field resulted in 4 in 6 patients and 5 in 2 patients of PSPT, while that was 3 in 2 patients, 4 in 12 patients, and 5 in 2 patients of IMPT.

Table 1 shows all parameters of PSPT and IMPT, and Table 2 shows p values comparing PSPT and IMPT when the irradiation field is shifted. In PSPT, *D*_max_, *D*_mean_, and *D*_min_ were 103.3 ± 0.9, 99.8 ± 0.4, and 90.8 ± 5.6% of prescription dose, respectively. CI and INH were 0.46 ± 0.08 and 0.1 ± 0.03, respectively. In IMPT, that was 103.6 ± 0.6, 99.9 ± 0.1 and 94.9 ± 1.3%, and 0.48 ± 0.08 and 0.07 ± 0.01, respectively. *D*_min_ of IMPT was higher than that of PSPT (*p* = 0.01). INH of IMPT was lower than that of PSPT (*p* = 0.004) (Table 1).

**Table 1:** Dose comparison of IMPT and PSPT

	PSPT	IMPT	*p* value
*D*_max_	103.3 ± 0.9%	103.6 ± 0.6	0.315
*D*_mean_	99.8 ± 0.4%	99.9 ± 0.1	0.307
*D*_min_	90.8 ± 5.6%	94.9 ± 1.3	0.01
CI	0.46 ± 0.08	0.48 ± 0.08	0.488
INH	0.1 ± 0.03	0.07 ± 0.01	0.004

*D*_max, mean, min_ values are percentages of the prescribed dose

**Table 2:** Difference of IMPT and PSPT for the movement of the fields

	mm	− 5	− 4	− 3	− 2	− 1	0	1	2	3	4	5
C												
*D*_max_	AP	0.407	0.475	0.520	0.617	0.688	0.315	0.802	0.882	0.968	0.969	0.898
	Lat	0.495	0.474	0.499	0.666	0.721	0.315	0.769	0.701	0.669	0.631	0.606
	CC	**0.000**	**0.000**	**0.005**	0.943	0.349	0.315	0.523	**0.047**	**0.008**	**0.004**	**0.003**
*D*_mean_	AP	0.362	0.348	0.465	0.496	0.533	0.307	0.609	0.641	0.680	0.690	0.791
	Lat	0.481	0.495	0.474	0.538	0.537	0.307	0.574	0.556	0.580	0.560	0.535
	CC	**0.001**	**0.002**	**0.007**	**0.043**	0.156	0.307	0.883	0.355	0.183	0.094	0.059
*D*_min_	AP	**0.008**	**0.008**	**0.009**	**0.009**	**0.009**	**0.010**	**0.009**	**0.010**	**0.010**	**0.010**	**0.011**
	Lat	**0.009**	**0.009**	**0.009**	**0.010**	**0.010**	**0.010**	**0.010**	**0.010**	**0.012**	**0.014**	**0.016**
	CC	**0.000**	**0.000**	**0.001**	**0.002**	**0.004**	**0.010**	**0.015**	**0.019**	**0.021**	**0.034**	0.060
CI	AP	0.482	0.493	0.502	0.531	0.522	0.488	0.548	0.596	0.613	0.586	0.599
	Lat	0.437	0.461	0.467	0.491	0.510	0.488	0.557	0.562	0.555	0.544	0.597
	CC	0.124	0.174	0.245	0.346	0.464	0.488	0.674	0.844	0.976	0.838	0.767
INH	AP	**0.002**	**0.002**	**0.002**	**0.001**	**0.001**	**0.004**	**0.001**	**0.001**	**0.001**	**0.001**	**0.001**
	Lat	**0.003**	**0.002**	**0.002**	**0.002**	**0.001**	**0.004**	**0.001**	**0.002**	**0.002**	**0.003**	**0.005**
	CC	**0.001**	**0.000**	**0.000**	**0.001**	**0.001**	**0.004**	**0.001**	**0.001**	**0.001**	**0.003**	**0.007**
L												
*D*_max_	AP	0.701	0.693	0.698	0.689	0.694	0.315	0.732	0.750	0.750	0.742	0.735
	Lat	0.213	0.324	0.504	0.623	0.739	0.315	0.705	0.605	0.483	0.361	0.294
	CC	0.541	0.532	0.549	0.586	0.616	0.315	0.934	**0.028**	**0.000**	**0.000**	**0.000**
*D*_mean_	AP	0.499	0.485	0.533	0.518	0.533	0.307	0.574	0.594	0.626	0.653	0.635
	Lat	0.450	0.519	0.522	0.592	0.562	0.307	0.559	0.573	0.544	0.507	0.563
	CC	0.341	0.341	0.391	0.405	0.439	0.307	0.680	0.864	0.966	0.907	0.894
*D*_min_	AP	**0.009**	**0.009**	**0.009**	**0.009**	**0.009**	**0.010**	**0.009**	**0.009**	**0.009**	**0.009**	**0.009**
	Lat	**0.017**	**0.015**	**0.012**	**0.010**	**0.009**	**0.010**	**0.009**	**0.010**	**0.012**	**0.015**	**0.022**
	CC	**0.000**	**0.001**	**0.002**	**0.007**	**0.009**	**0.010**	**0.009**	**0.009**	**0.010**	**0.010**	**0.010**
CI	AP	0.473	0.482	0.500	0.506	0.563	0.488	0.565	0.583	0.599	0.610	0.617
	Lat	0.535	0.531	0.539	0.531	0.548	0.488	0.549	0.553	0.578	0.795	0.793
	CC	0.502	0.511	0.540	0.513	0.523	0.488	0.561	0.572	0.536	0.517	0.513
INH	AP	**0.001**	**0.002**	**0.001**	**0.001**	**0.001**	**0.004**	**0.001**	**0.001**	**0.001**	**0.001**	**0.001**
	Lat	**0.007**	**0.005**	**0.002**	**0.002**	**0.001**	**0.004**	**0.001**	**0.002**	**0.003**	**0.005**	**0.009**
	CC	**0.010**	**0.003**	**0.001**	**0.002**	**0.001**	**0.004**	**0.001**	**0.001**	**0.002**	**0.006**	**0.021**

Number means *p* value. Bold means significant change

When the irradiation field of the C level was shifted, *D*_max_ was lower in IMPT than PSPT at the level of more than 3 mm to the caudal direction and more than 2 mm to the cranial direction (*p* = 0.000–0.047). *D*_mean_ was higher in IMPT at the range of more than 2 mm to the caudal direction (*p* = 0.001–0.043). *D*_min_ was higher in IMPT at all levels in all directions except 5 mm to the cranial direction (*p* = 0.000–0.034). CI tended to decrease in PSPT when the irradiation field was shifted to the caudal direction. INH was lower in IMPT at all levels in all directions (*p* = 0.000–0.007) (Table 2).

**Fig. 1:**
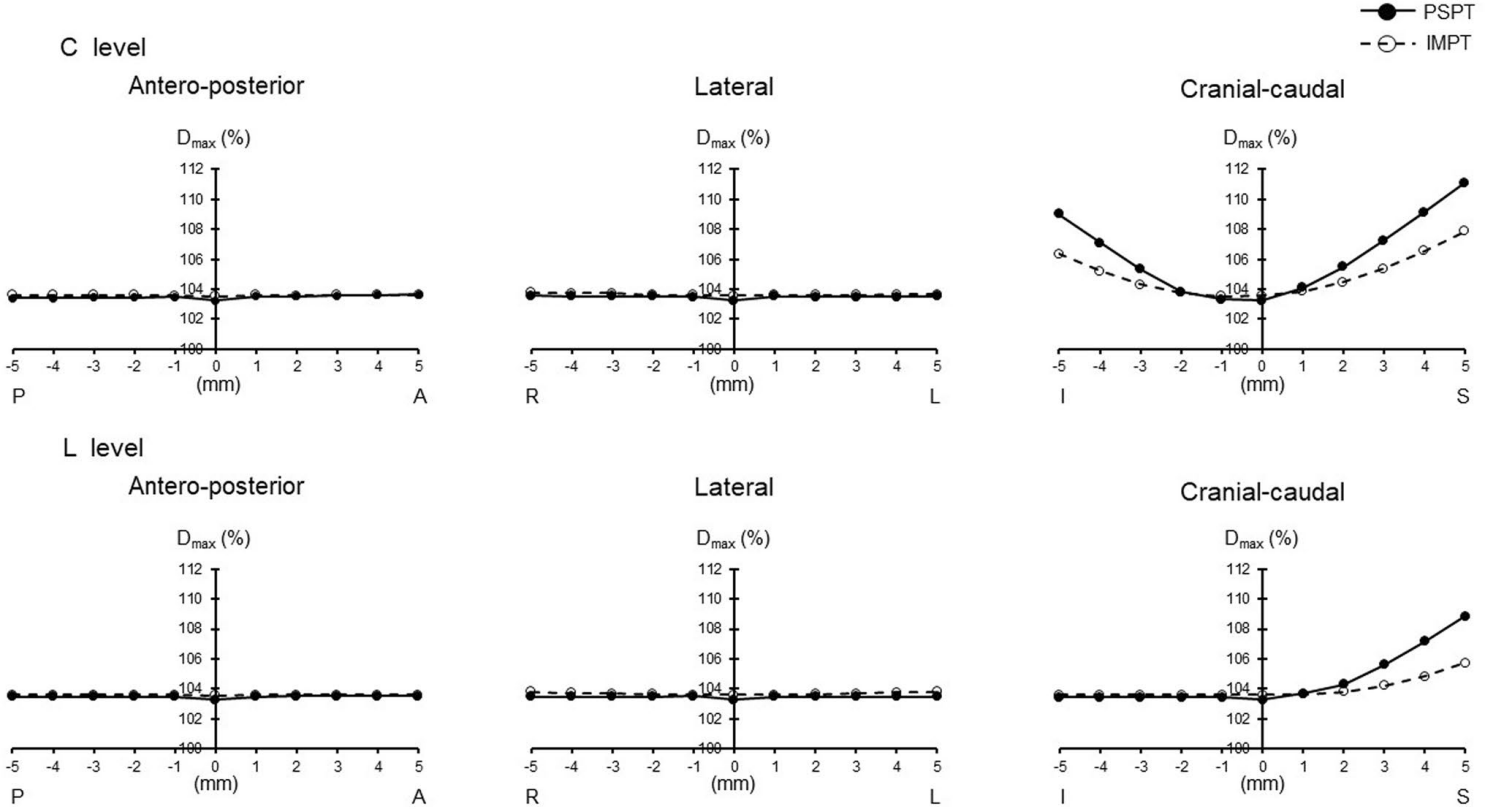
PTV dose change (*D*_max_). *P* posterior, *A* anterior, *R* right, *L* left, *I* inferior, *S* superior. Black circle with solid line: PSPT. White circle with dot line: IMPT. All data represent mean value

**Fig. 2:**
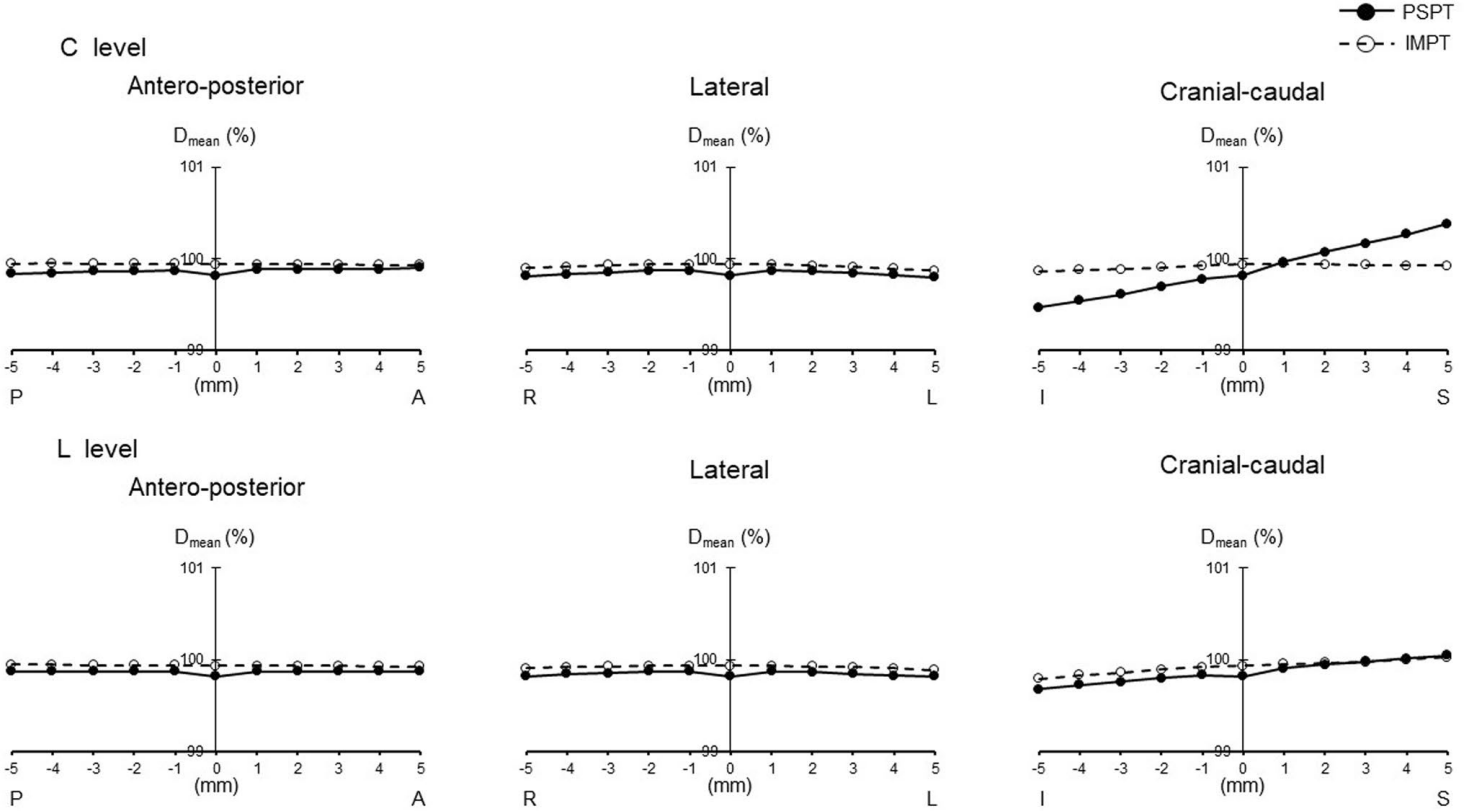
PTV dose change (*D*_mean_)

**Fig. 3:**
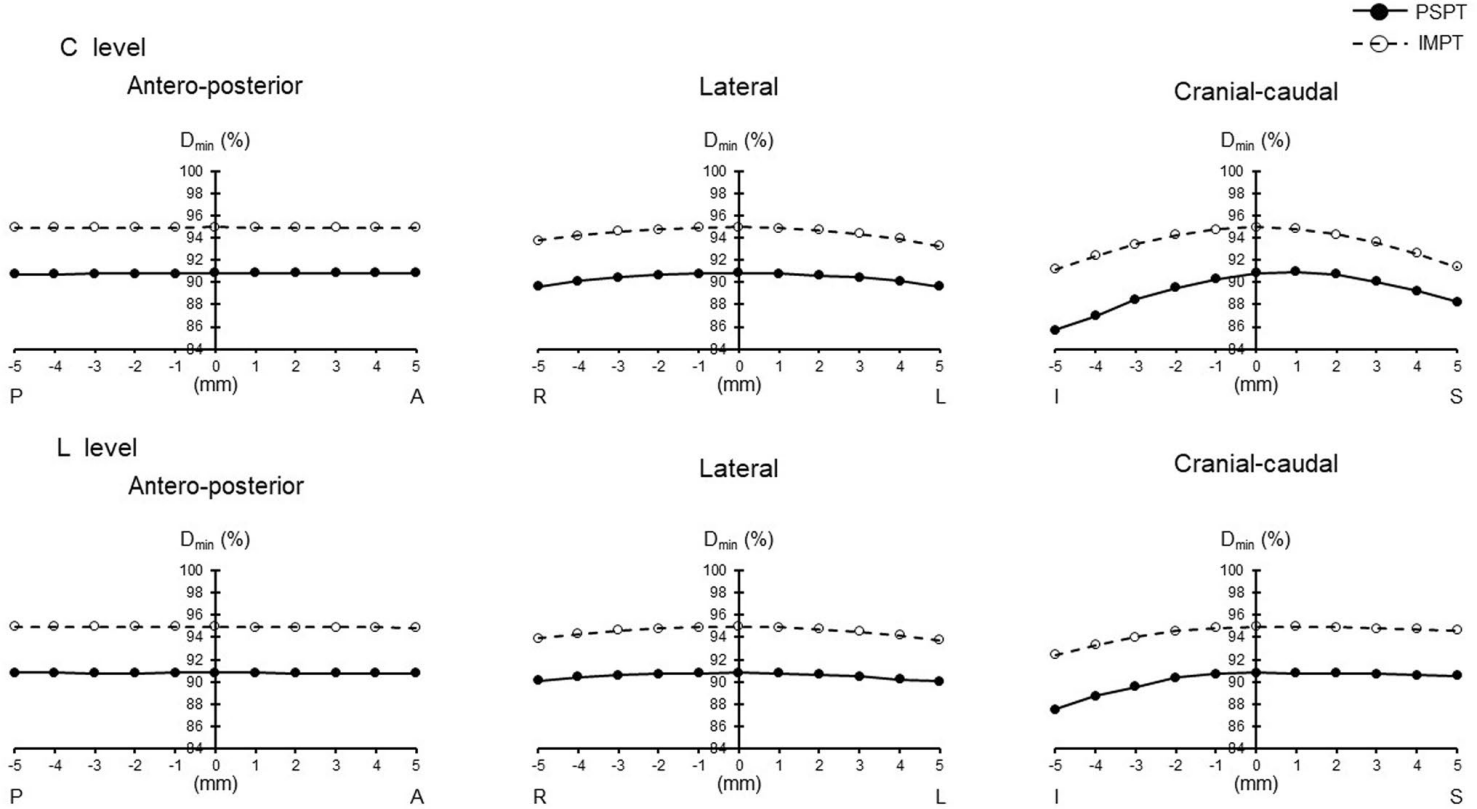
PTV dose change (*D*_min_)

**Fig. 4:**
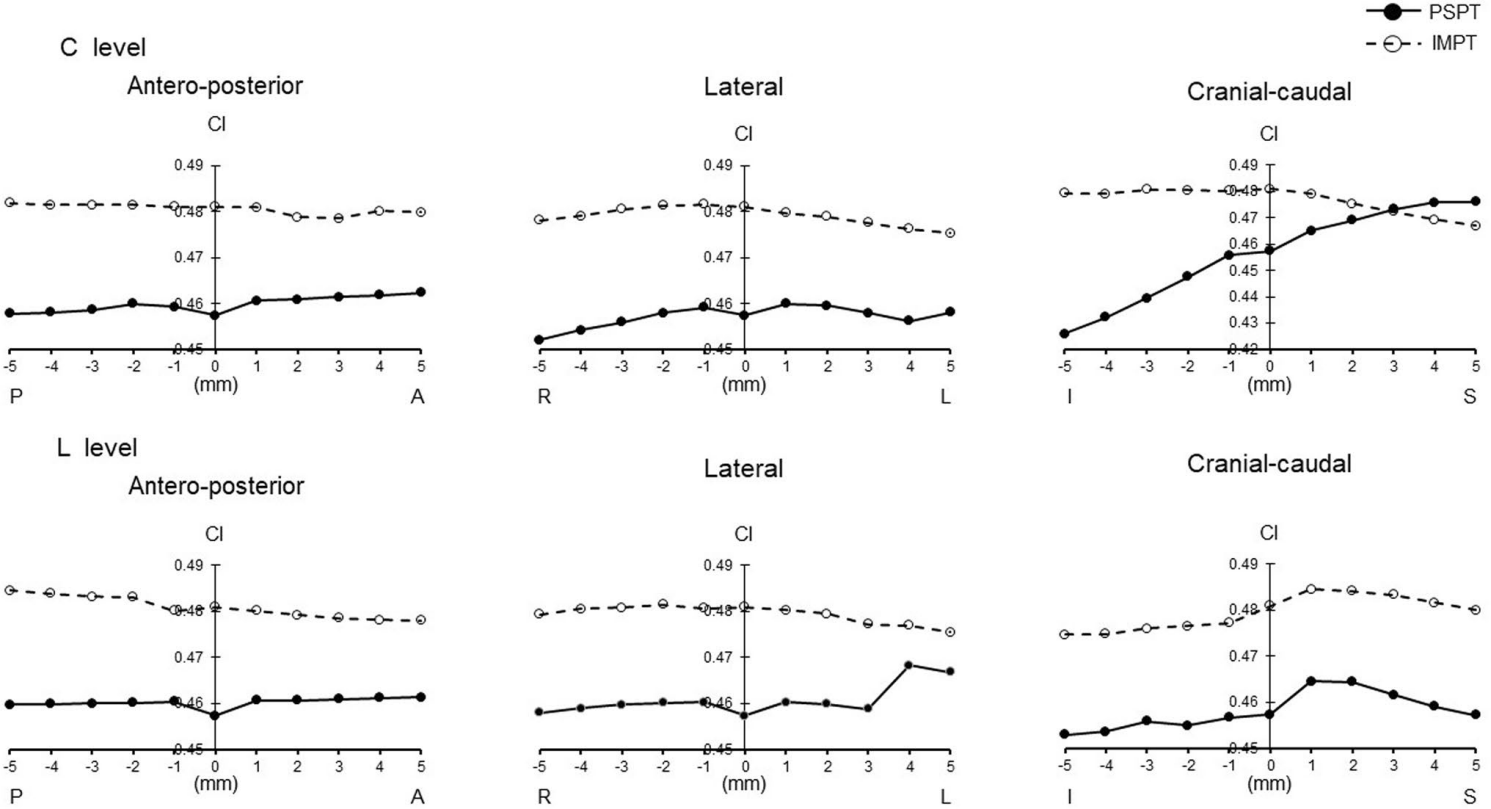
PTV dose change (CI)

**Fig. 5:**
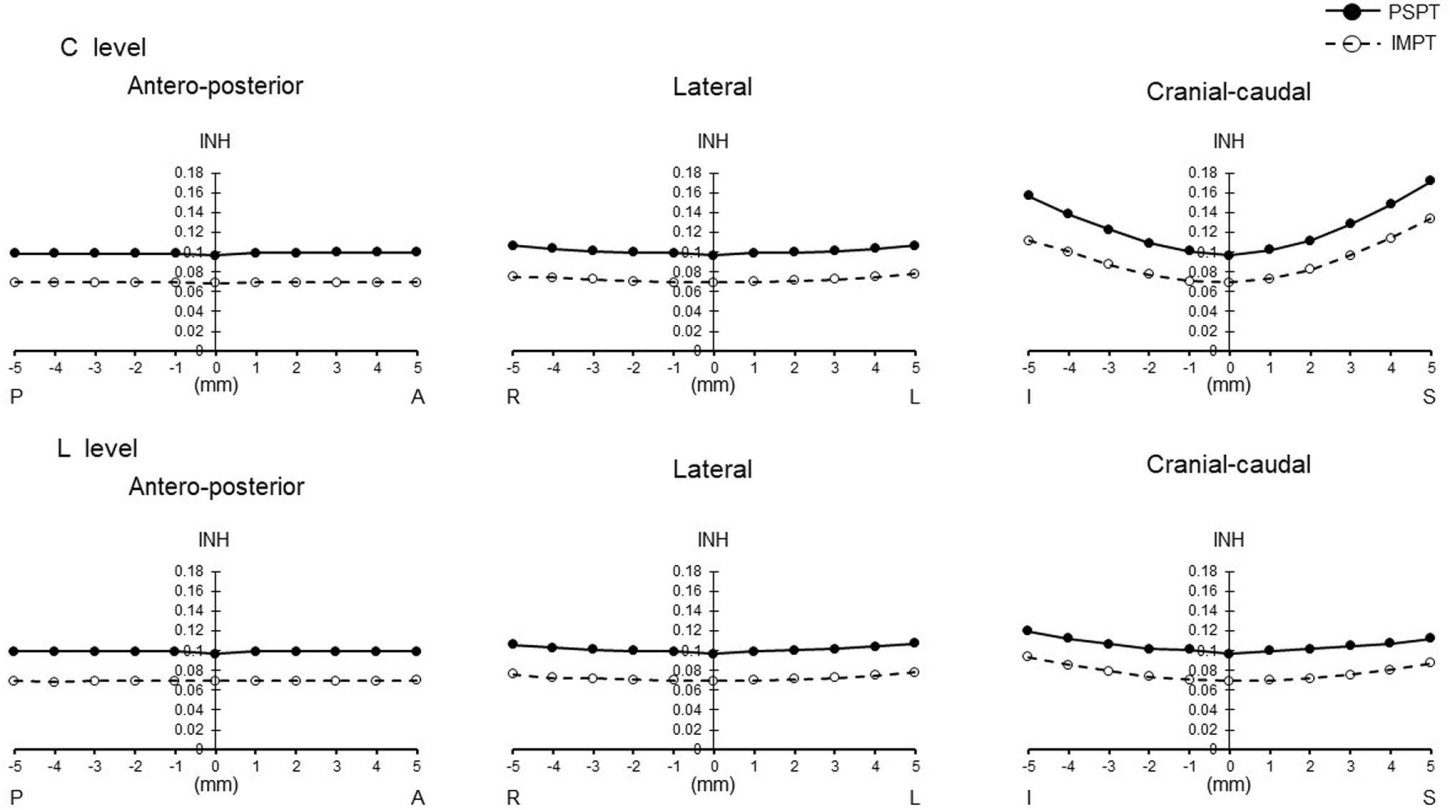
PTV dose change (INH)

Figures 1, 2, 3, 4, and 5 show the changes in all PSPT and IMPT parameters when the C and L level irradiation fields are shifted in six directions within a range of 5 mm. When the irradiation field of the L level was shifted, *D*_max_ was lower in IMPT at the level of more than 2 mm to the cranial direction (*p* = 0.000–0.028). *D*_mean_ was almost same in all directions. *D*_min_ was higher in IMPT at all levels in all directions (*p* = 0.000–0.022). CI showed similar trend in all directions. INH was lower in IMPT at all levels in all directions (*p* = 0.001–0.021) (Figs. 1, 2, 3, 4, 5).

## Discussion

IMPT offers excellent flexibility in terms of adjusting the available irradiation field and dose shaping. IMPT can accomplish increased dose conformity and increased degrees of freedom in dose shaping capabilities. Giantsoudi et al. investigated a simulation study and revealed that dose conformity of IMPT was better than PSPT [18]. Stoker et al. reported IMPT softened the field edge gradient for junction fields [20]. In our study, *D*_min_ was higher and INH was lower in IMPT, which was similar to the past studies.

In IMPT, there is no definite length required for field junction. Each facility has to decide the length of junction by comprehensively considering the irradiation field size, smoothness of dose distribution, etc. Stoker et al. reported the length of the junction was ideally 10 cm and minimum of 6 cm was proposed [20]. Fellin et al. also reported they chose the length of the junction around 6–8 cm [21]. In our facility, junction length decided as 7 cm considering smooth dose gradation, field size, and number of fields.

Although we do not believe there is a clinically significant difference between the target doses for PSPT and IMPT, the minimum dose for PSPT of 90.8% of the prescribed dose seems somewhat inadequate; for PSPT, the minimum PTV dose was less than 95% in all eight cases, due to Bragg peak width, MLC, and bolus parameters to comply with OAR dose constraints and dose reduction at field junctions. In particular, irradiation dose of cribriform plate and dose constraint of the lenses were struggled in many cases. In addition, dose reduction at the field junction occurred in cases where the brain and C level junction had to be set at the cerebellar level. We believe that IMPT can easily overcome these problems. As shown in the results, PTV dose stability associated with set-up error about the antero-posterior and lateral directions was stable for both PSPT and IMPT. This means margin and robustness parameters setting is adequate enough. On the other hand, cranial–caudal direction movement was different. IMPT was more stable than PSPT for both C level and L level migration in the cranio-caudal direction. For example, assuming a C level shift, Dmax changed from 104.1 to 111.1% (difference: + 7%) for PSPT, whereas it changed less in IMPT, from 103.3 to 106.7% (difference: + 3.4%); Dmin changed from 90.8 to 85.7% (difference: − 5.1%) for PSPT, but was similarly little changed from 94.9 to 91.2% (difference: − 3.7%) for IMPT. It is reasonable to assume that the dose gradient at the IMPT junction helps mitigate the abrupt dose changes associated with set-up errors. This difference is clearly evident in the change in CI, which changed from 0.43 to 0.48 (difference: + 0.05) for PSPT but hardly changed at all for IMPT, from 0.48 to 0.47 (difference: − 0.01). Patterns of change varied by site. *D*_max_ change of C level showed similar trend, no matter which direction it shifts up or down. It means that *D*_max_ is almost the same whether the irradiated field at the C level overlaps with the brain or with the thoracic spinal cord level. On the other hand, since there is no overlap in the downward movement at L level, it is a natural result that there is no change unlike the upward movement. For the same reason, it is reasonable that *D*_min_ shows a similar trend for up and down movement at C level, but no change for upward movement at L level with no loss of boundary area. Both *D*_max_ and *D*_min_ changed more steeply with cranio-caudal movement at the C level than at the L level, which can explain why INH tends to be more unstable at the C level than at the L level.

In this study, we evaluated robustness with PTV. Unlike adults who can fine-tune their own body position, in sedated children, even if iso-centers are adjusted accurately, there is often a large shift at the margins of the irradiation field due to body twisting and slouching. In the case of CSI, the deviation is even greater because multiple irradiation fields (usually 4–5 fields) are irradiated by sliding the patient's iso-center. Therefore, we analyzed the robustness of PTV because CTV evaluation is not always clinically meaningful and a wider range of evaluation is needed. In the daily clinic, set-up error is adjusted to be within 3 mm in our facility. However, for the same reason as above, the irradiated area may be deviate more than that in the peripheral region. Taking this into consideration, the range of shift in this study was set at 5 mm.

Stoker et al. reported IMPT can achieve dose variations < 5% in comparison with the 25% dose variation observed for PSPT for a 2 mm per field set-up error [20]. Fellin et al. investigated the dose change in case the field was shifted using IMPT [21]. They found the maximum CTV dose increased when the beams were shifted toward each other along the cranial–caudal direction, while the target coverage worsened when the beams were apart along the same direction. Our data are quite similar to the result of past studies. At our facility, we believe we are able to match positions to within 3 mm accuracy during routine treatment. Calculating from our own data, we can infer that the irradiation dose of the PTV is accurate to within maximum 1.7% and minimum 0.6% at the C level, and maximum 1.6% and minimum 1% at the L level.

PSPT was used until Oct 2019 and IMPT has been used since then in our facility. When our facility was first established, only broad-beam irradiation was available, so in actual clinical practice, while treating patients with broad-beam irradiation, we were also preparing for scanning irradiation at the same time. IMPT is not only superior to PMPT in dose stability, but also eliminates the need to create a compensator. When the preparation for scanning irradiation was completed, the treatment of CSI was changed from PSPT to IMPT. We plan to use IMPT for all future CSI treatments.

Some limitations are included in this study. The sample size is by no means sufficient. So far, there are no findings suggesting adverse events due to overdose or recurrence due to insufficient dose. However, there is no way to increase the number of PSPT cases further since the usual practice has already been shifted from PSPT to IMPT. We plan to accumulate IMPT cases and study its efficacy and safety in more number of patients.

## Conclusion

The PTV doses of IMPT and PSPT are robust and stable in both anterior–posterior and lateral directions at both C level and L level, but IMPT is more robust and stable than PSPT for cranial–caudal movements.
